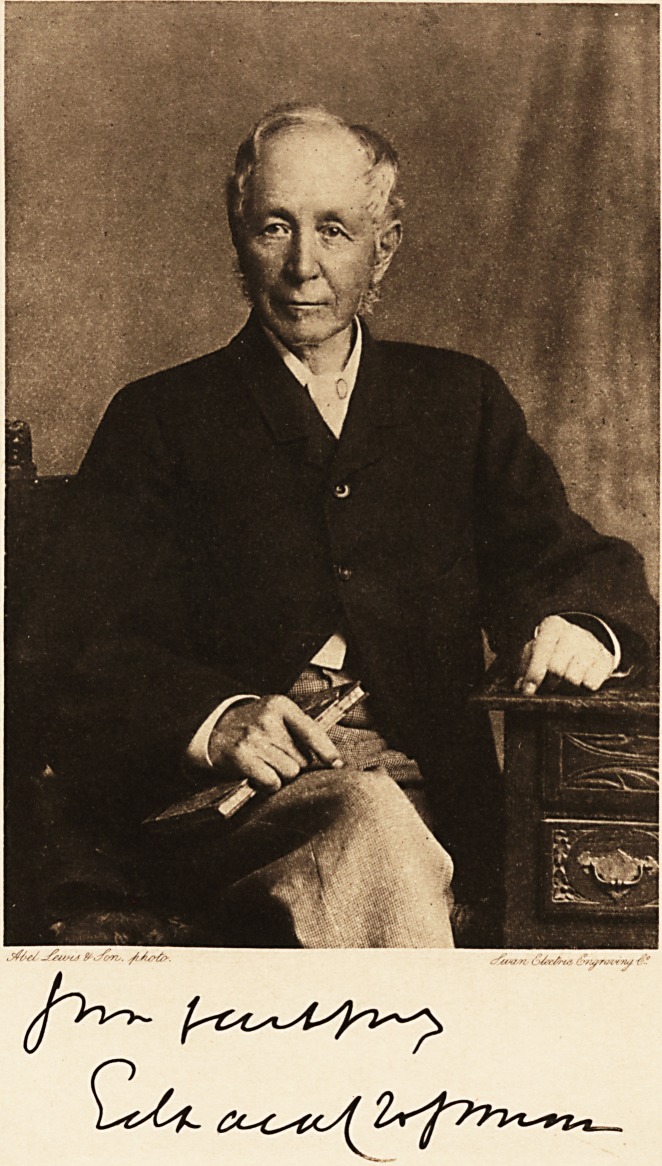# Edward Crossman

**Published:** 1904-09

**Authors:** 


### The Bristol Medico-Chirurgical Journal.

"*Scire est nescire, nisi id me*
*Scire alius sciret.*"

SEPTEMBER, 1904.

### Obituary.

### EDWARD CROSSMAN, M.D.

It is with much regret we record the death of Edward Crossman, of White's Hill, Hambrook, Bristol. Dr. Crossman was born at Lower Hazel, Alveston, Gloucestershire, on September 30th, 1832. His death took place somewhat suddenly, though not unexpectedly, at his residence at White's Hill on September 1st, 1904, at the age of 72, owing to an attack of dyspnœa connected with heart disease. A Gloucestershire man, of a good old family and highly connected, the name of Crossman is well known. His loss will be mourned by a large circle of medical and lay friends.

Dr. Crossman received his professional education in London at the London Hospital, where eventually he held the important post of House Surgeon; having qualified in 1855 as M.R.C.S. and L.S.A., while in 1869 he obtained the L.R.C.P. Lond. He further pursued his studies in Paris, as was not an uncommon thing at that time for a student to do.

He began his life's work at Hambrook, where he came as assistant to Mr. John Hay, a surgeon of the old school, well known and thought much of in the neighbourhood. He shortly afterwards entered into partnership with Mr. Hay in 1856, and eventually succeeded to the practice. Dr. Crossman in this way was a link between the old school of practitioners who freely bled and blistered their patients, cupped them, leeched them, and used the seton, well represented in Mr. John Hay, and the newer school of medicine and surgery with its more carefully thought-out and applied practice.

His bright and cheery disposition was united to an intellectual power of no mean order, with a zeal and true devotion to his work. His thought and care over his cases was full of deep anxiety that no necessary point should be lost sight of, but due weight given it in the interest of the patient's well-being and the issue of the case. To this was added the greatest kindness, consideration, and sympathy. It need hardly be added that he was one who was entirely qualified for the practice of his art, as well as one whose opinion was highly valued by his medical brethren around. He was an excellent surgeon and a good operator.

He became Medical Officer of Health to Barton Regis District in 1886, where he did good work. He was Senior Surgeon to the Frenchay General Dispensary, and Medical Officer to the Diocesan Training College, Fishponds, where his loss will be much felt. He also gave his gratuitous services to St. Michael's Orphanage at Frampton Cotterell. It will be remembered that in 1883 he was President of the Bath and Bristol Branch of the British Medical Association.

When Mr. Albert Napper, of Cranleigh, started the idea of cottage hospitals, Dr. Crossman was greatly interested in the matter, and saw Mr. Napper several times about it. Having made himself well acquainted with the details of the subject, he set to work himself to start such a hospital in his own district. He, his family and friends were successful in their efforts, and the Hambrook Village Hospital was opened in 1867 as a memorial of his work in this direction. It has been highly valued in the neighbourhood and proved itself a most useful institution. What Dr. Crossman and his family have done for the institution, or the great good that has been effected by it, none hardly but themselves know.

Dr. Crossman, notwithstanding all the work that was entailed by his large practice and by his other many interests in life, showing how great his physical and mental powers were, undertook the arduous task of going up to Durham in 1886 and taking its M.D. degree: not long after this his son, Dr. Frank Crossman, joined him in the practice.

Dr. Crossman took deep interest in other matters than those of his profession. He was an ardent Freemason, and for many years a most regular and interested attendant at the meetings and other duties of his lodge.

Dr. Crossman was also a loyal and staunch Churchman, and took an intelligent and deep interest in Church matters, being a member of the Diocesan Conference for many years, and a member of the House of Laymen. He was one of those chiefly interested in obtaining the formation of the Ecclesiastical District of Winterbourne Down, as well as the building of the Church of All Saints and the Vicarage. From the commencement he was churchwarden, and was rarely absent from his post at the chief services of the church. He was a manager of the Parish Schools, which owe much to his supervision. All Saints, Winterbourne Down, will lose in him one who has been most intimately bound up in its development and welfare as a parish.

With such an activity of mind and body, it cannot be surprising to find he was an ardent politician—an active supporter of the Primrose League and of the Member of Parliament. On such occasions his speeches were of a high order and very telling with his audiences.

Medical men of his time took less notice of the need of rest than is now considered right. Dr. Crossman, though no holiday-taker, still was a keen sportsman. He was an excellent shot, enjoying a day's shooting over dogs in the old-fashioned style, while not entirely opposed to more modern ways. He was at such times full of pleasant fun, and enjoyed the jokes of his fellow-guns after a day's shooting in Somersetshire or amongst the grouse and the oats at Llanthony Abbey.

The loss of such a man will fall heavily on his family, but the loss will not be theirs alone. The clergy, the parish workers, his relatives, friends, patients, and fellow-parishioners will all feel his loss, and their sympathy and mourning will be a touching testimony of the worth in which he was held in the thoughts and affections of his fellow-men amongst whom he lived and worked in so many ways.

It has been said that a lengthy obituary notice might be abbreviated as follows: "He was a good man, for he loved his garden." The garden was a hobby of Crossman's. He had a great love for his flowers, as well as for his vegetables and fruit. A good deal of his spare time was spent in it and in the tree-margined, beautiful little valley through which the River Frome wanders at the foot of the steep rocks on which his house of White's Hill is built. Advice had been given him, since his anxious condition had been known, not to exert himself in his work or to climb the steep slopes in his grounds. But he had done it all his life, and it was difficult to tear himself from it. Perhaps some over-exertion of this sort on the evening of September 1st excited the fatal attack which brought his honoured career to an end. Thus it can be said he died as no doubt he would have wished—at work until the end—and now he lies in God's garden at rest and in peace.

### LAC VINUM INFANTUM:
### A REVIEW OF THE WORK OF INFANT MILK DEPÔTS.

BY

J. M. Fortescue-Brickdale, M.A., M.D. Oxon.,

## Figures and Tables

**Figure f1:**